# Supplementary material for: Acquisition of Streptomycin Resistance by Oxidative Stress Induced by Hydrogen Peroxide in Radiation-Resistant Bacterium Deinococcus geothermalis
Source: Int J Mol Sci. 2022 Aug 28;23(17):9764. doi: 10.3390/ijms23179764 (PMC9456066; doi:10.3390/ijms23179764)
Supplement: Supplementary file 1 [file ijms-23-09764-s001.zip › ijms-1847768-supplementary.pdf]

Supplementary data

Table S1. List of used primer sequences.

Primer	Sequence (5' > 3')	Gene
Construction of mutants		
0257_LF_*KpnI*	AAGGTACCTATCGCCATAAGCTCTAA	*dps3*
0257_LR_*SalI*	AAGTCGACGCTCCAATTGTGGATTAA	*dps3*
0257_RF_*XbaI*	AATCTAGAAAGAACTCCTTGACGTCGT	*dps3*
0257_RR_*PstI*	AACTGCAGGGTTTTGGAAACTGCTTTA	*dps3*
0281_LF_*BamHI*	AAGGATCCAATCAGGATCACATTCAGTC	*dps1*
0281_LR_*BglII*	AAAGATCTGACTTTCTTCACGCTTGCG	*dps1*
0281_RF_*KpnI*	AAGGTACCATGAACTGAGCGGGG	*dps1*
0281_RR_*SalI*	AAGTCGACGCTTTCTCATAGCAAACC	*dps1*
2840_ LF_*KpnI*	AAG GTA CCC GGG TGA ACA TCA GAT	*lysR*
2840_ LR_*SalI*	AAG TCG ACC AGA ATC AAT TCG TCC T	*lysR*
2840_ RF_*XbaI*	AAT CTA GAC TAC AGA CGA GAA CGG T	*lysR*
2840_ RR_*PstI*	AAC TGC AGG TCT TAA GCC TCG GA	*lysR*
To check for streptomycin related genes		
Dgeo_2335_F	TGATGGCGGTGGTTGTCTT	*rsmG*
Dgeo_2335_R	ATTTTCAGTGGATGGCGCG	*rsmG*
Dgeo_1873_F	GAACACCTTCAGCGGCTCTT	*rpsL*
Dgeo_1873_R	CCCTCCGGCATCCTTGC	*rpsL*
Dgeo_0447_F	AGGGTCACTTTCCTGGTCTGT	*mthA*
Dgeo_0447_R	GGCGAGCCACTGGTTGGAA	*mthA*
Dgeo_0776_F	GACCCATCCCGACCACTGA	*mthA*
Dgeo_0776_R	GCAGGCCTGGTCCTTCACA	*mthA*
Dgeo_0534_F	AGGTGATATTGCGAGTGCGT	*znuA*
Dgeo_0534_R	AGACGATGGTGGTGAACGC	*znuA*
Dgeo_0915_F	CATGAGTGACAACATCGGCG	*nuoG*
Dgeo_0915_R	TGTACGCAAAGGTCGTCAGC	*nuoG*
Dgeo_1583_F	CGGAGCGTACTTTGAGTGG	*trkH*
Dgeo_1583_R	CATCTCATAGAGGGTGGTCG	*trkH*
16S_rRNA_F1	GCGCAAGCTTGGATTAAACG	*dgeo*_R0002
16S_rRNA_R1	CAGCTCCCAAAGCGCACA	*dgeo*_R0002
16S_rRNA_F2	GACAGGGTGGTACACGCG	*dgeo*_R0008
16S_rRNA_R2	GCGGGAAATGCAGCAAGATG	*dgeo*_R0008
16S_rRNA_F3	GATCATTGTGCTGGGGGAC	*dgeo*_R0013
16S_rRNA_R	GGCGCAAGTGACGATGACA	*dgeo*_R0013
16S_rRNA_F4	GAAGCAAAGGGCCACCTGA	*dgeo*_R0067
16S_rRNA_R4	CGGTGTGTGTACCCGTCT	*dgeo*_R0067
Dgeo_1841_F	TGGGATTCCCGATGGTGAC	*rpsD*

Dgeo_1841_R	AGAAGAAGTTCCGCGCCTAAA	*rpsD*
Quantitative Real Time PCR		
Dgeo_1202_F	AGCTCGGTGCGGTAGGTAAA	*aadA*
Dgeo_1202_R	CGTTCGGGTATCTCGAGGG	*aadA*
Dgeo_0646_F	TGAAGGTAATCGCTGCCGT	EF-Tu
Dgeo_0646_R	GCAAAAGGAACGTTTGAGCG	EF-Tu
Dgeo_1869_F	GTGAAGGTAATCGCTGCTGT	EF-Tu
Dgeo_1869_R	CAAAAGGAACGTTTGAGCGGA	EF-Tu

Figure legends

Figure. S1. Comparison of growth pattern among wild-type, Δ*dgeo*_0257, and Δ*dgeo*_0281 parent and Sm-resistant mutants in antibiotics-free TGY medium (A) and TGY+Sm 50 μg/mL medium (B).

Figure. S2. Amino acid alignment of RpsL (ribosomal protein S12) among *E. coli*, *D. geothermalis*, and *T. thermophilus* (A). Two streptomycin interacting regions were marked red color such as P42, K43, K44, and P45, and K88 and 91P amino acid residues. Scheme indicates streptomycin interacting region using Mol* viewer (Sehnal *et al.*, 2021) in 30S small ribosomal subunit from PDB 4DV7 (B). The decoding center region composed of ribosomal proteins S12, S4, and helices h18, h27, and h44 of 16S rRNA. Light green indicates the streptomycin interacting surface.

Figure. S3. PCR detection of additional Sm-resistant related genes such as *znuA* (*dgeo*_0534), *nuoG* (*dgeo*_0915), *trkH* (*dgeo*_1583) and *dgeo*_1841 for ribosomal protein S4. Lanes: M, size marker; 1, wild-type; 2-5, WT S1-S4; 6, Δ*dgeo*_0257 S1; 7-9, Δ*dgeo*_2840 S1, S2, and S*rsmG*; 10, Δ*dgeo*_0281 S1.

Figure. S4. PCR detection of four 16S rRNAs. Lanes: M, size marker; 1-4, wild-type S1-S4**;** 5 Δ*dgeo*_0257 S1; 6-8, Δ*dgeo*_2840 S1, S2, and S*rsmG*; 9, Δ*dgeo*_0281 S1.

Figure. S5. Differential antibiotics-resistant phenotypes from complement strain. Streptomycin-resistant MIC levels among wild-type, Δ*dgeo*_2840, and a complement (C2840) (A). Nine streptomycin-resistant mutants from the complement strains (C2840) exhibit different streptomycin MIC values following K43R, K88R, K88E, and P91H comparing with parent strains (B).

Figure. S6. Phenotypic recovery of cell growth in complement strain. Both Δ*dgeo*_2840/pRADgro and Δ*dgeo*_2840/pRADgro::*dgeo*_2840 were grew on TGY medium containing 3 μ g/mL of chloramphenicol.

Figure. S7. Phenotypic recovery of hydrogen peroxide sensitivity in complement strain. Wild-type, Δ*dgeo*_2840, and a complement (C2840) strains were cultivated till OD_{600} 2.0, then exposed 80-120 mM hydrogen peroxide of final concentration for one hour. Then the diluted samples were spotted on TGY agar plates and incubated for 2 days at 48 °C.

Figure. S1

A

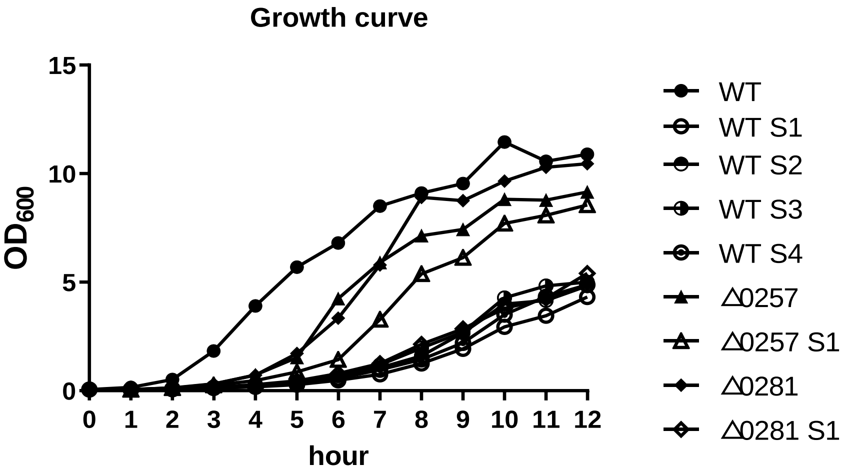
Growth curve
OD600
15
10
5
0
0 1 2 3 4 5 6 7 8 9 10 11 12
hour
WT
WT S1
WT S2
WT S3
WT S4
△0257
△0257 S1
△0281
△0281 S1

B

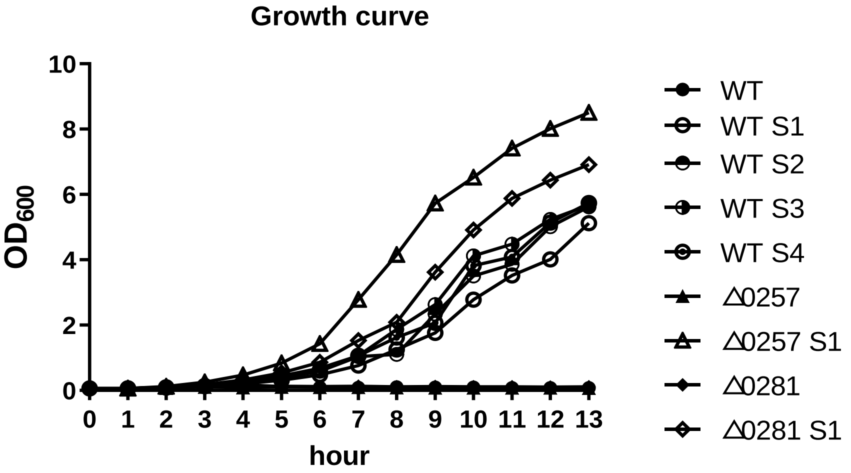
Growth curve
OD600
10
8
6
4
2
0
0 1 2 3 4 5 6 7 8 9 10 11 12 13
hour
WT
WT S1
WT S2
WT S3
WT S4
△0257
△0257 S1
△0281
△0281 S1

Figure. S2A

```
             1                                                         60
                                                   43
Dgeo         MPTTQQLLRKGRTTLQKKSKVPALKGSPFRRGVCTVVKTTTPKKPNSALRKIARVRLSSG
Tth          MPTINQLVRKGREKVRKKSKVPALKGAPFRRGVCTVVRTVTPKKPNSALRKVAKVRLTSG
Ecoli        MATVNQLVRKPRARKVAKSNVPALEACPQKRGVCTRVYTTTPKKPNSALRKVCRVRLTNG
             *.* :**:** *      **:****:..* :***** * *.***********:.:***:.*

             61                                                       120
                                        88 91
Dgeo         FEVTAYIPGEGHNLQEHSVVLIRGGRVKDLPGVRYHIVRGSLDTQGVKDRNKSRSKYGTK
Tth          YEVTAYIPGEGHNLQEHSVVLIRGGRVKDLPGVRYHIVRGVYDAAGVKDRKKSRSKYGTK
Ecoli        FEVTSYIGGEGHNLQEHSVILIRGGRVKDLPGVRYHTVRGALDCSGVKDRKQARSKYGVK
             :***:** ***********:**************** ***  *  *****:::*****.*

             121         134
Dgeo         KPKAGAAAAGAKKK
Tth          KPKEAAKTAAKK—
Ecoli        RPKA----------
             :**
```

Figure. S2B

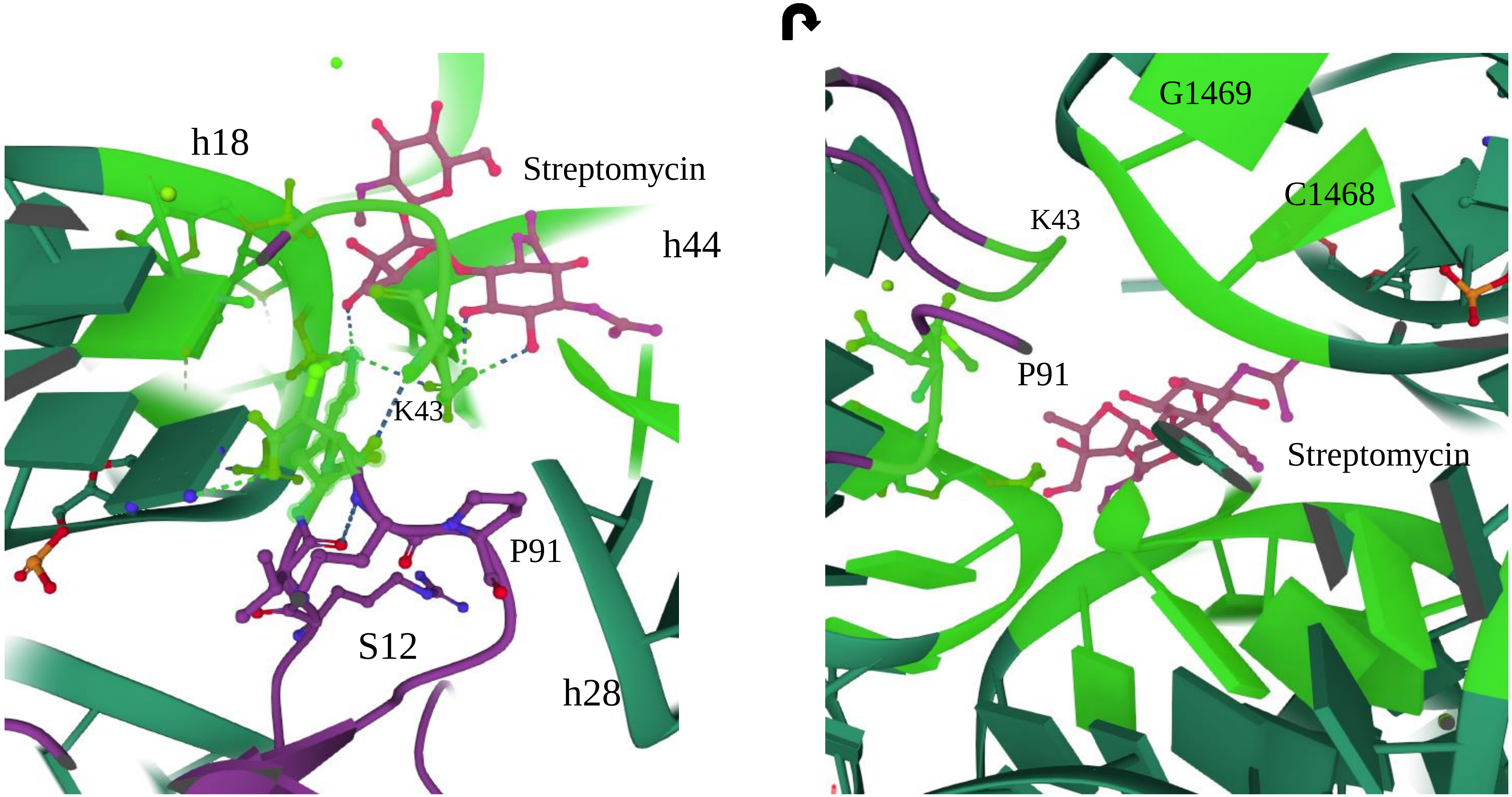

Resource : PDB 4DV7 (*T. thermophilus* 30S subunit)

Figure. S3

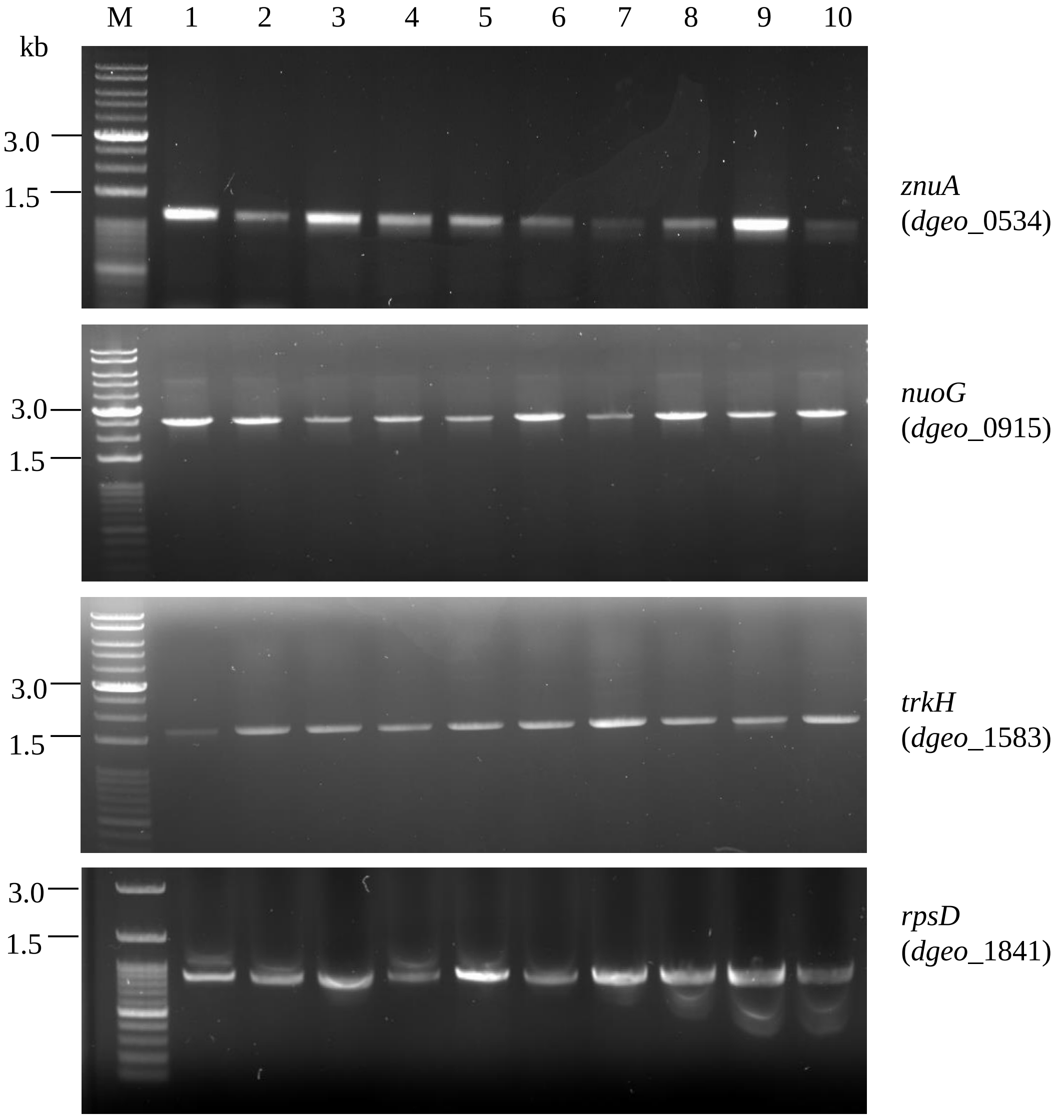
M 1 2 3 4 5 6 7 8 9 10
kb
3.0
1.5
znuA
(dgeo_0534)
3.0
1.5
nuoG
(dgeo_0915)
3.0
1.5
trkH
(dgeo_1583)
3.0
1.5
rpsD
(dgeo_1841)

Figure. S4

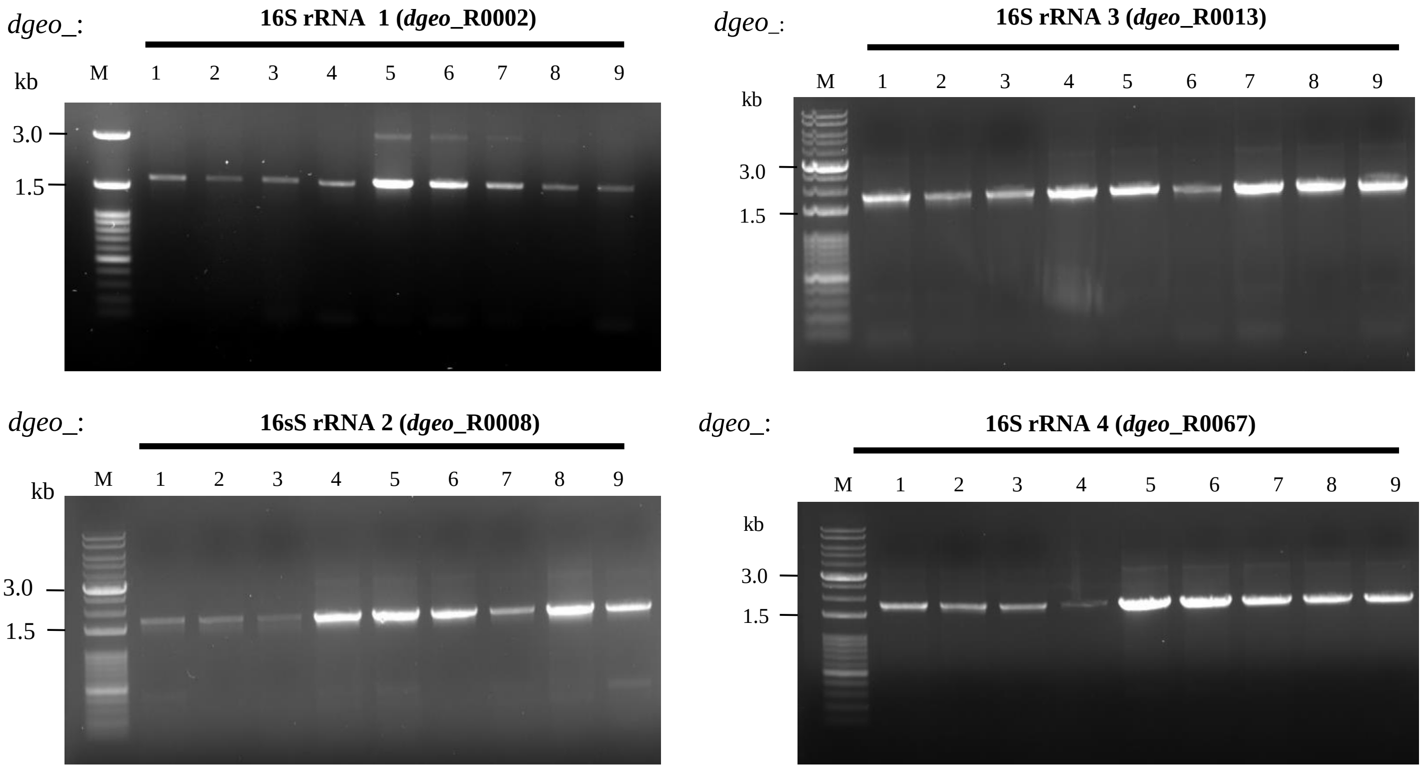

dgeo_:
16S rRNA 1 (dgeo_R0002)
kb
M 1 2 3 4 5 6 7 8 9
3.0
1.5
dgeo_:
16S rRNA 3 (dgeo_R0013)
kb
M 1 2 3 4 5 6 7 8 9
3.0
1.5
dgeo_:
16sS rRNA 2 (dgeo_R0008)
kb
M 1 2 3 4 5 6 7 8 9
3.0
1.5
dgeo_:
16S rRNA 4 (dgeo_R0067)
kb
M 1 2 3 4 5 6 7 8 9
3.0
1.5

Figure. S5

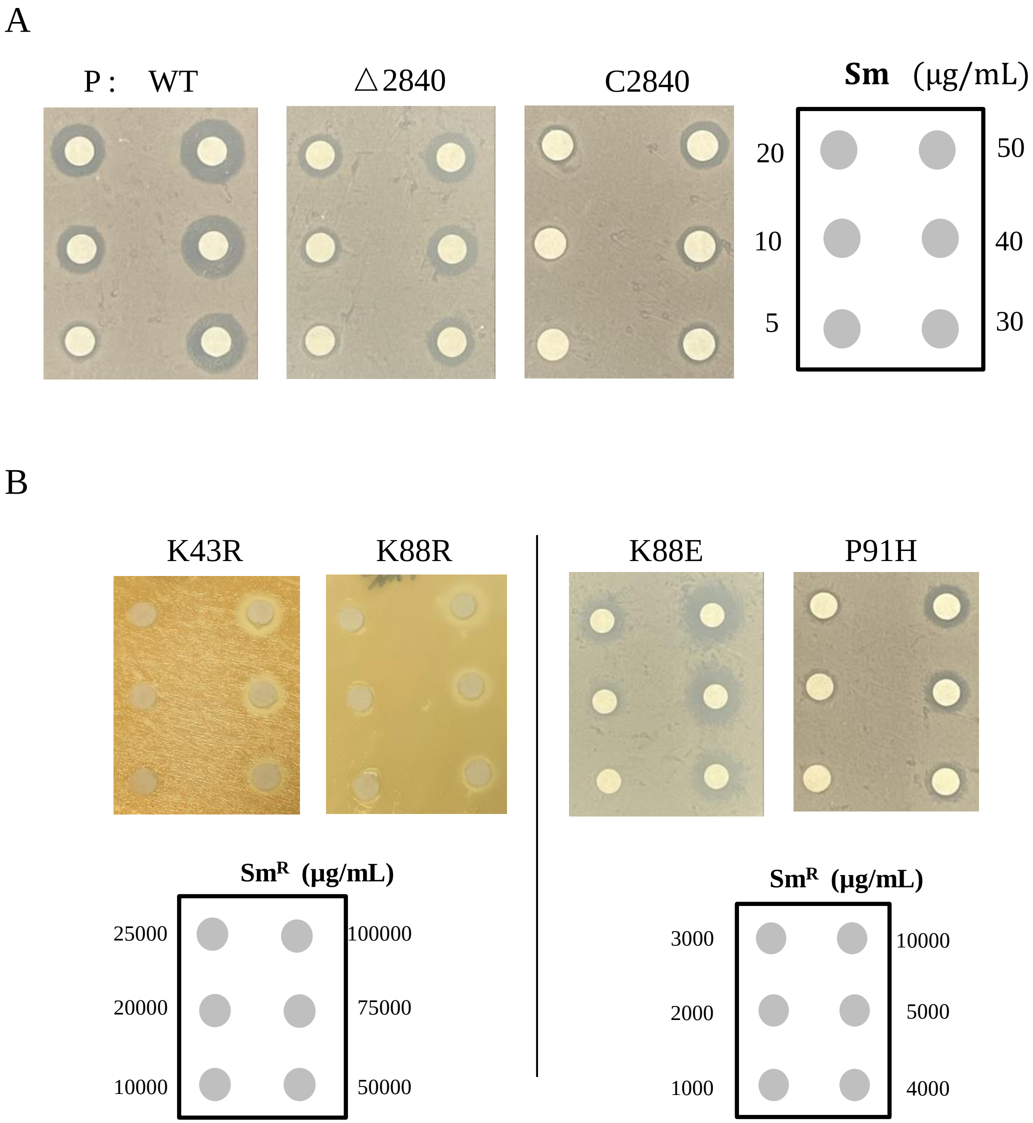

A
P : WT
△2840
C2840
Sm (μg/mL)
20
10
5
50
40
30
B
K43R
K88R
K88E
P91H
SmR (μg/mL)
25000
20000
10000
100000
75000
50000
SmR (μg/mL)
3000
2000
1000
10000
5000
4000

Figure. S6

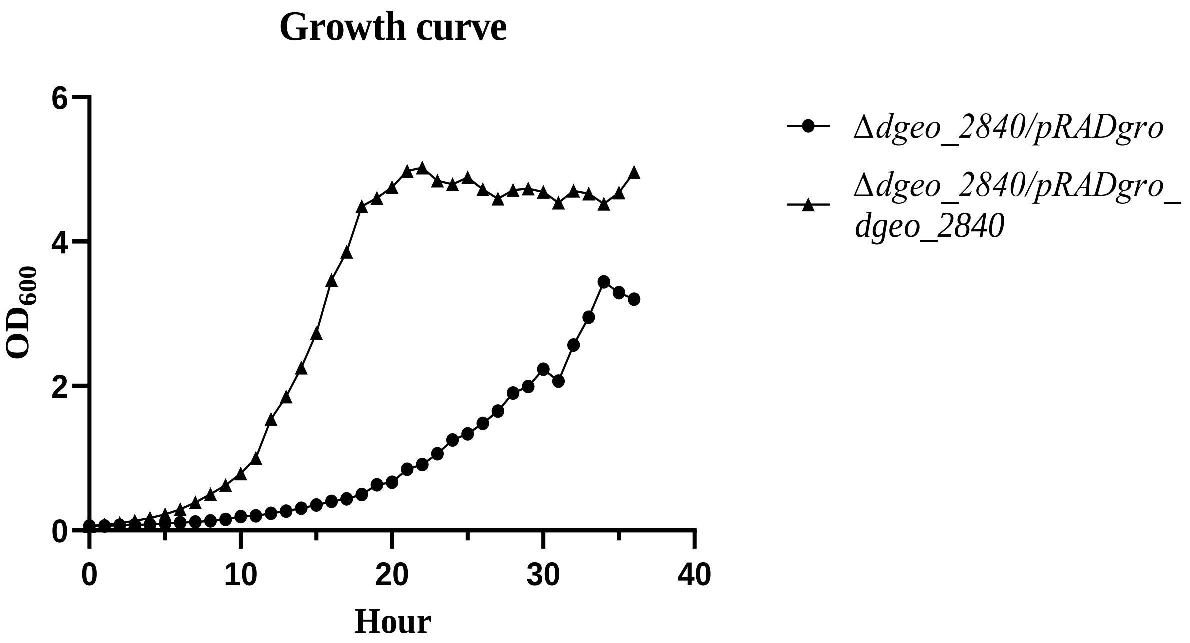
Growth curve
6
4
2
0
OD600
0
10
20
30
40
Hour
Δdgeo_2840/pRADgro
Δdgeo_2840/pRADgro_
dgeo_2840

Figure. S7

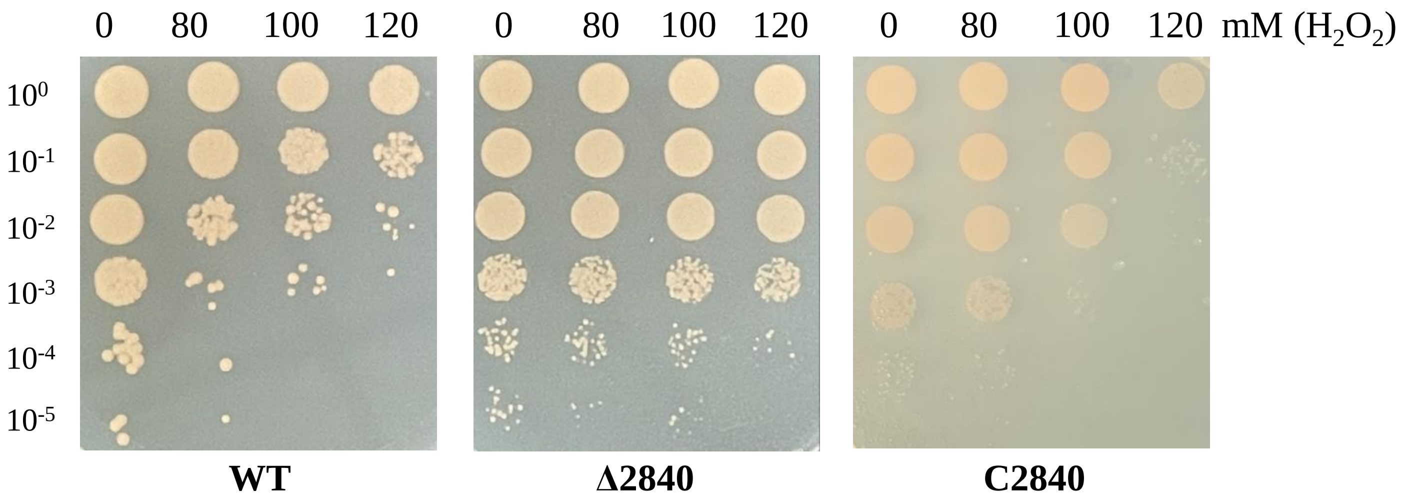
0 80 100 120
0 80 100 120
0 80 100 120 mM (H_2O_2)
10^0
10^{-1}
10^{-2}
10^{-3}
10^{-4}
10^{-5}
WT
Δ2840
C2840
